# CNMa–CNMa receptor at microbiome–gut–brain axis: novel target to regulate feeding decision

**DOI:** 10.1038/s41392-021-00708-y

**Published:** 2021-07-25

**Authors:** Liyuan Peng, Hai-Yan Yin, Canhua Huang

**Affiliations:** 1 School of Basic Medical Sciences, Chengdu University of Traditional Chinese Medicine, Chengdu, China; 2 State Key Laboratory of Biotherapy and Cancer Center, West China Hospital, and West China School of Basic Medical Sciences & Forensic Medicine, Sichuan University, and Collaborative Innovation Center for Biotherapy, Chengdu, China; 3 International Collaborative Centre on Big Science Plan for Purinergic Signalling, Chengdu University of Traditional Chinese Medicine, Chengdu, China; 4 Acupuncture and Chronobiology Key Laboratory of Sichuan Province, Chengdu, China

**Keywords:** Physiology, Neuroscience

**Fig. 1:**
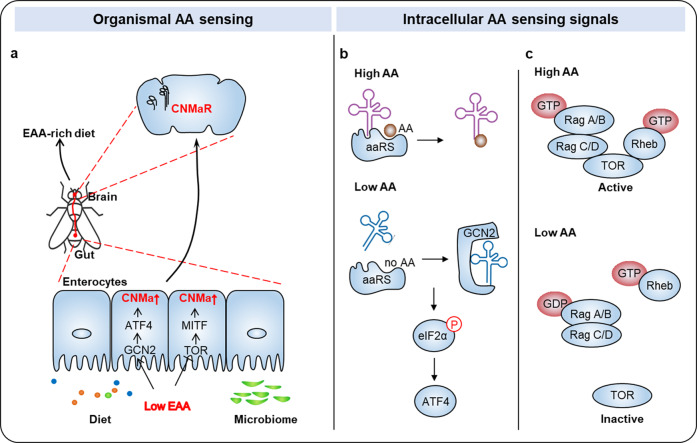
Amino acid-sensing machineries**. a** During the process of amino acid-driven food selection, the gut communicates with the brain through neurons, secreted hormones and neurotransmitters, and the gut microbiome. In this study, the authors find that gut enterocytes detect and respond to protein limitation through upregulating CNMa expression and communicating with CNMaR-expressing neurons in the brain, leading to the compensatory appetite for L-EAAs. AA, amino acids. **b** GCN2, an indirect amino acid sensor, could detect any low-level amino acid through binding to the uncharged cognate tRNA, which leads to a conformational change of GCN2. This structural alteration activates the kinase activity of GCN2, culminating in inhibitory phosphorylation of eukaryotic translation initiator factor 2 α (eIF2α) and the increased transcription of starvation-relevant genes, such as activating transcription factor 4 (ATF4). aaRS, aminoacyl transfer RNA synthetase. **c** TOR is positively regulated by the GTP-charged form of the Ras-like GTPase Rheb (Rheb-GTP) and heterodimeric Rag GTPase. Amino acids deprivation suppresses the kinase activity of TOR through inhibiting the interaction of TOR with the Ras-like GTPase Rheb (Rheb-GTP) and the heterodimeric Rag GTPase.

A recent study published in *Nature* by Kim et al. discovers that the CNMamide (CNMa)–CNMa receptor (CNMaR) axis plays a crucial regulatory role in response to deprivation of diet- and microbiome-derived amino acid in *Drosophila*.^1^ In detail, gut enterocytes detect and respond to protein limitation through upregulating CNMa expression and communicating with CNMaR-expressing neurons in the brain (Fig. 1). This microbiome–gut–brain axis stimulates a compensatory appetite for essential amino acids (EAAs), thereby restoring protein homeostasis. These findings highlight a previously uncharacterized role of CNMa–CNMaR neuronal signaling pathway in food selection.

For free-living animals, one prerequisite for survival is the ability to regulate the amount and types of food they consume in response to nutritional fluctuation. Protein is one of the most indispensable macronutrients, an inadequate intake of which may lead to multiple diseases, such as malnutrition. It has been previously described that dietary restriction of proteins results in an increased appetite for foods containing abundant protein or EAAs in animals. In this study, Kim et al. found that amino acid-deprived flies exhibited a strong preference for metabolizable L-EAAs over unmetabolizable D-EAAs.^1^ This finding further suggested that insufficient consumption of amino acids stimulates the ingestion of flies for L-EAAs. However, the detailed mechanism connecting a dietary protein deficiency and food selection, as well as the organ(s) or cell type(s) involved in this process are largely unexplored.

Organisms have evolved powerful machineries to detect a deficit of amino acids and trigger adaptive responses (Fig. 1). At the organismal level, starvation of amino acids leads to a changed conformation of dopaminergic neurons and an increased sensitivity of IR76b-expressing neurons, accompany by altered food preference.^2^ In addition, during the process of amino acid-driven food selection, the gut communicates with the brain through neurons, secreted hormones, and neurotransmitters. General control nonderepressible 2 (GCN2)- and target of rapamycin (TOR)-mediated signaling pathways represent two evolutionarily conserved intracellular mechanisms responsible for the accurate sensing of amino acid levels.^3^ More intriguingly, independent pieces of evidence suggested that these two amino acid-sensing pathways are able to guide dietary selection through the cross-talk between neuronal circuits.

In this study, the authors provide novel insights into the sophisticated regulatory network of food selection in *Drosophila*.^1^ Upon food consumption, enterocytes at the R2 segment of the anterior midgut efficiently monitor the abundance of L-EAAs. When EAAs are scarce in the internal milieu, enterocytes upregulated the expression of CNMa peptide, which potently activates CNMaR-expressing neurons in the anterior gastrointestinal tract and the brain.^1^ In addition to foods rich in protein, the gut microbiome is another possible resource of L-EAAs. Herein, the authors noted that specific gut commensal bacterium containing key genes for branched-chain amino acids (BCAAs) biosynthesis could provide the basal source of EAAs, while bacterial strains incapable of synthesizing BCAAs could not supply efficient EAAs for flies. Enterocytes are also able to adapt to a deficiency of microbiome-derived amino acid through expressing CNMa peptides. Furthermore, the authors proved that during EAAs deprivation, the inter-organ cross-talk of intestinal CNMa and neuronal CNMaR mediates the compensatory appetite for L-EAAs. CNMa, which can selectively and potently activate the orphan GPCR, CG33696 (CNMaR), was identified as a novel cyclic neuropeptide by computational analysis of the *Drosophila* genome in 2014. The following researches revealed that this peptide also exists in members of other species and that CNMa may function as modulators in neural and reproductive systems. Moreover, a series of high-throughput sequencing data have suggested that CNMa may be involved in the regulation of physiological activities, including *Drosophila* life span and ant social organization. However, the specific biological function and the underlying mechanism of CNMa remain undetermined.

Here, the authors demonstrate that CNMa serves as a key information transmitter in controlling feeding behavior in *Drosophila*. In addition, the regulatory mechanism of CNMa expression level is discovered for the first time: the expression level of CNMa is upregulated through the GCN2-ATF4 and TOR-MITF pathways, among which ATF4 and MITF possibly function as the transcription factors of CNMa. Recently, another study focused on the neural mechanisms integrating temperature sensation and sleep also showed that CNMa released by posterior dorsal neuron 1 (DN1p) neurons inhibits the Dh44-positive pars intercerebralis (PI) neurons to promote wakefulness through CNMaR, further validating the critical role of CNMa in transferring intercellular information. The amino acid-sensing pathway discovered in *Drosophila* resembles the mechanism underlying the adaptive response to protein deficiency in mammals. For example, in mammals, dietary protein restriction induces the expression of hepatic FGF21, which is similar to CNMa in *Drosophila*, through ATF4 and transcription factor EB pathways.^5^ The metabolic hormone FGF21 then communicates with the brain thus initiating a series of homeostatic responses.

*Drosophila* CNMa is expressed in multiple organs, including the fat body and the brain. However, tissue-specific experiments showed that only CNMa in the enterocytes was upregulated during protein starvation.^1^ Future investigation may focus on the specificity of enterocytes in monitoring amino acid levels. Another remaining question is that whether CNMa reacts with intestinal CNMaR-expressing neurons to convey information to the brain or directly activates CNMaR-expressing neurons in the brain. Recent studies illustrated a direct interplay between the enteric neurons and the brain, providing evidence that CNMa may transmit the low-protein status to the brain through CNMaR-expressing enteric neurons. Nevertheless, it is equally possible that released CNMa disseminates in the circulation system and directly stimulates the brain, which requires further validation.

As an organ directly exposed to the ingested food, the gut consists of several types of cells, including enterocytes, stem cells, and enteroendocrine cells, which coordinately orchestrate feeding behavior. Surprisingly, enterocytes in the *Drosophila* have been generally considered to serve a role in nutrients digestion and absorption, while enteroendocrine cells cope with nutritional stress and maintain nutritional homeostasis through conveying the message in the form of neuropeptides to the brain. Thus, this study revealed a brand new function of enterocytes as a nutrient sensor, which could sense and respond to the deprivation of even a single EAAs through releasing a gut hormone. In concert with this, another recent work reported that enterocytes served as controllers of food intake for larval development under nutrient limitation.^4^ These findings provide clues for previously unrecognized functions of enterocytes and advance our understanding of the complex system for feeding control.

It has been identified in *Drosophila* that some of the amino acid sensors, for instance, female-specific independent of transformer (FIT) peptide and dopaminergic (DA) neurons, play a part in protein-specific hunger-induced feeding decisions. Here, this work demonstrated that CNMaR functions as a novel neuronal receptor for a gut-secreting hormone CNMa, which connects the microbiome–gut–brain axis thus regulating the shifting of food preference. Altogether, the discovery of the CNMa–CNMaR at microbiome–gut–brain axis provides important implications for understanding the elaborated regulatory network of nutrient homeostasis maintenance.
